# Loss of Von Hippel–Lindau (*VHL*) Tumor Suppressor Gene Function: *VHL*–HIF Pathway and Advances in Treatments for Metastatic Renal Cell Carcinoma (RCC)

**DOI:** 10.3390/ijms22189795

**Published:** 2021-09-10

**Authors:** Hyunho Kim, Byoung Yong Shim, Seung-Ju Lee, Ji Youl Lee, Hyo-Jin Lee, In-Ho Kim

**Affiliations:** 1 Division of Medical Oncology, Department of Internal Medicine, St. Vincent’s Hospital, The Catholic University of Korea, 93 Jungbu-daero, Paldal-gu, Suwon 16247, Korea; h2kim@catholic.ac.kr (H.K.); shimby@catholic.ac.kr (B.Y.S.); 2 Department of Urology, St. Vincent’s Hospital, College of Medicine, The Catholic University of Korea, 93 Jungbu-daero, Paldal-gu, Suwon 16247, Korea; seungju@catholic.ac.kr; 3 Department of Urology Cancer Center, Seoul St. Mary’s Hospital, The Catholic University of Korea, 222 Banpo-daero, Seocho-gu, Seoul 06591, Korea; uroljy@catholic.ac.kr; 4 Department of Internal Medicine, Chungnam National University School of Medicine, 266 Munhwa-ro, Jung-gu, Daejeon 35015, Korea; cymed@cnuh.co.kr; 5 Division of Medical Oncology, Department of Internal Medicine, Seoul St. Mary’s Hospital, The Catholic University of Korea, 222 Banpo-daero, Seocho-gu, Seoul 06591, Korea

**Keywords:** kidney cancer, tumor suppressor gene, *VHL*, HIF, VEGFR, immune checkpoint inhibitor

## Abstract

Renal cell carcinoma (RCC) is a malignancy of the kidney originating from the tubular epithelium. Inactivation of the von Hippel–Lindau tumor-suppressor gene (*VHL*) is found in most clear cell renal cell carcinomas (ccRCCs). The *VHL*–HIF–VEGF/VEGFR pathway, which involves the von Hippel–Lindau tumor suppressor protein (*VHL*), hypoxia-inducible factor (HIF), vascular endothelial growth factor (VEGF), and its receptor (VEGFR), is a well-studied therapeutic target for metastatic ccRCC. Therefore, over the past decade, anti-angiogenic agents targeting VEGFR have served as the standard treatment for metastatic RCC. Recently, based on the immunomodulatory effect of anti-VEGFR therapy, anti-angiogenic agents and immune checkpoint inhibitor combination strategies have also emerged as therapeutic strategies. These advances were made possible by the improved understanding of the *VHL*–HIF pathway. In this review, we summarize the historical evolution of ccRCC treatments, with a focus on the involvement of the *VHL*–HIF pathway.

## 1. Introduction

**Figure 1:**
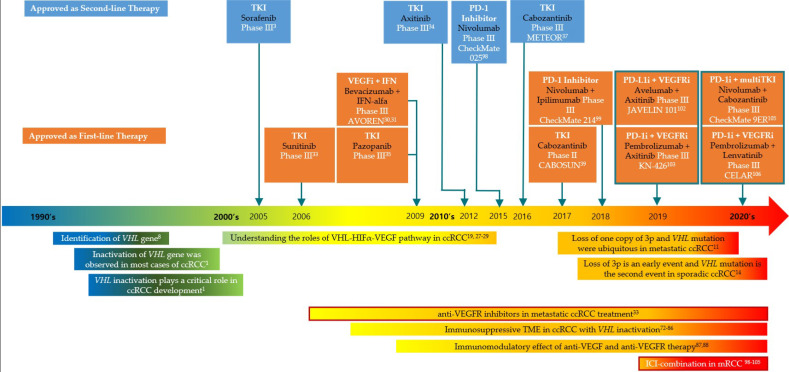
The *VHL*–HIF pathway and advances in treatments for metastatic ccRCC. Abbreviations: ccRCC, clear cell renal cell carcinoma; HIF, hopoxia-inducible factor; ICI, immune checkpoint inhibitor; mRCC, metastatic renal cell carcinoma; PD-1, programmed cell death protein 1; PD-1i, PD-1 inhibitor; PD-L1 programmed death-ligand 1; PD-L1i, PD-L1 inhibitor; TKI, tyrosine kinase inhibitor; TME, tumor microenvironment; VEGF, vascular endothelial growth factor; VEGFi, VEGF inhibitor; VEGFR, vascular endothelial growth factor receptor; *VHL*, von Hippel Lindau.

Renal cell carcinomas (RCCs), which account for more than 90% of kidney cancers, are malignant tumors originating from the epithelium of the renal tubules [1]. The most common histological subtype is clear cell renal cell carcinoma (ccRCC), which is found in approximately 85% of metastatic RCC (mRCC). Although nephrectomy can be used to treat localized RCC, metastasis develops in up to 30% of cases after radical surgery. Systemic therapy is the main treatment option for mRCC [1]. In the past, treatment with interleukin-2 (IL-2) and interferon-α (IFNα) was attempted in patients with mRCC, and it sometimes effected a durable response, but overall treatment outcomes were not satisfactory [2]. In 1993, the von Hippel–Lindau gene (*VHL*) was discovered in a patient with von Hippel–Lindau disease with RCC [1]. Follow-up studies in the 2000s elucidated the primary oncogenic pathway involved in ccRCC, and the new understanding of the roles of *VHL*-hypoxia inducible factor (HIF) and vascular endothelial growth factor (VEGF) gave rise to improved treatment options for metastatic ccRCC [1]. Treatment now hinges primarily on anti-angiogenic tyrosine kinase inhibitors (TKIs), which target the vascular endothelial growth factor receptor (VEGFR). Sorafenib, the first targeted treatment for advanced ccRCC, entered phase III trials in 2005 [3]; it was followed by sunitinib, pazopanib, axitinib, and cabozantinib [4,5]. The next era of ccRCC treatment commenced in recent years with a strategy combining immune checkpoint inhibitors (ICIs) with anti-angiogenic TKIs. This strategy works because the blockade of VEGF-A/VEGFR2 plays a role in immune modulation by affecting tumor vasculature, immune cell infiltration, and cytokine levels [6]. Recently, the improved understanding of metabolic changes and treatment resistance in the hypoxic tumor microenvironment (TME) allowed for the development of HIF inhibitors. Clinical trials related to this are currently being conducted in patients with mRCC. Taken together, it is apparent that improved understanding of the *VHL*–HIF pathway has led to these promising advances in mRCC treatment. This review aims to summarize the evolution of treatments for metastatic ccRCC as they relate to the *VHL*–HIF pathway (Figure 1). 

## 2. *VHL* and the Loss of Chromosome 3p in ccRCC Tumorigenesis

The term *VHL* was first used in the early 1900s when Eugen von Hippel and Arvid Lindau described retinal angiomatosis and hemangioblastoma of the cerebellum and spinal cord, respectively; the term *VHL* disease was first used in 1936 to describe a disease of inherited hypervascular tumors [7]. *VHL* was first identified in 1993 by Latif et al., who used a site-cloning method in a patient with *VHL* disease [8]. *VHL* is a tumor suppressor gene that regulates cell division, cell death, cell differentiation, and response to cell stress, and it is compatible with Knudson’s two-hit theory of cancer causation [7]. *VHL* is located on the short arm (3p25) of chromosomal 3, and loss of at least one copy of chromosome 3p is observed in over 90% of sporadic ccRCCs [9,10]. It is well known that biallelic *VHL* loss plays a critical role in ccRCC development [11]. Regarding tumor heterogeneity, genomic analyses of multi-region tumor samples from mRCC patients showed that *VHL* mutation and loss of one copy of 3p were ubiquitous in all analyzed regions and tumors. In contrast, other driver mutations, such as *MTOR*, *PTEN*, *SETD2*, and *KDM5C,* exhibited intra-tumor heterogeneity [12]. Moreover, through recent whole-genome and whole-exome sequencing analyses of *VHL* wild-type ccRCC tumors, hot spots of mutation in *TCEB1* were found [13]. *TCEB1* encodes the general transcription elongation factor, elongin C, which is required for the functional operation of the *VHL* E3 ubiquitin ligase complex [13]. These findings suggest that defective *VHL*-mediated signaling is not only a common feature of ccRCC, but actually the most important factor in the pathogenesis of ccRCC. 

Additionally, according to the TRACERx Renal study, in which ccRCC biopsies were collected from 33 patients using a multi-region sampling approach, loss of 3p usually occurs first through chromothripsis, with *VHL* inactivation as a second event due to either *VHL* mutation or the hypermethylation of the *VHL* promoter region [14]. The loss of 3p as the first event typically occurs 5–20 years before tumor diagnosis, and *PBRM1*, *BAP1*, and *SETD2,* which are commonly observed in other mutated genes in sporadic ccRCC, are coincidentally located on chromosome 3p [14]. This confers the probability that the inactivation of *PBRM1*, *BAP1*, or *SETD2* can also occur during the tumorigenesis of ccRCC, similar to *VHL*. Consequently, although *VHL* is the main player in the pathobiology of ccRCC, these other tumor suppressor clusters are also likely to be involved; in fact, recent studies have shown that *VHL* inactivation alone is not sufficient for the development of ccRCC. However, the fact that *VHL* inactivation is a critical event in the tumorigenesis of ccRCC is not disputed. As descriptions of tumor suppressor genes other than *VHL* are beyond the scope of this review, we recommend that interested readers refer to de Cubas and Rathmell’s excellent 2018 review article [15].

## 3. *VHL* Inactivation and Targeted Therapy in ccRCC

Since *VHL* disease presents with hypervascular tumors such as hemangioblastoma and RCC, researchers have conducted studies on the relationship between *VHL* and VEGF, a pro-angiogenic molecule; such associations have also been reported in non-hereditary RCC [16,17]. In 1996, a ccRCC cell line study reported that both VEGF mRNA and protein were overexpressed in cases of *VHL* loss, and dysregulated VEGF was recovered when wild-type *VHL* was reintroduced [18]. Furthermore, *VHL*–HIF–VEGF signaling was described in a study in which the re-expression of *VHL* restored the oxygen-dependent instability of HIFα [19]. These studies conferred a theoretical background for the development of anti-angiogenic treatments.

### 3.1. The *VHL*–HIF–VEGF Pathway

The main substrate for the *VHL* protein is HIFα, which plays a key role in the cellular response to hypoxia [19]. HIF was first identified as a hypoxia-activated transcription factor that binds to the human erythropoietin gene enhancer in low-oxygen conditions [20]. Structurally, HIF comprises a heterodimer composed of an alpha and beta subunit; three subtypes (HIF1, HIF2, and HIF3) have been identified. Proline residues (P564) of HIF1α and HIF2α are hydroxylated by the prolyl hydroxylase domain enzyme (PHD). As dioxygen is required as a co-substrate in the hydroxylation process [21,22], the hydroxylation of HIFα occurs under normal-oxygen conditions but not in low-oxygen conditions. Meanwhile, *VHL* combines with the transcriptional elongational factors elongin B and C. Elongin C binds to the Cullin2–Rbx1 complex to form a *VHL* E3 ubiquitin ligase complex [23]. This complex has the ability to ubiquitinate proteins, such as hydroxylated HIFα, marking them for degradation. Consequently, hydroxylated HIFα is polyubiquitylated by the *VHL* E3 complex under normal-oxygen conditions [22,24,25], and HIFα is finally degraded by the 26S proteasome through the interaction between Tat-binding protein-1 (TBP1) and the β subunit of *VHL* [26]. On the other hand, in hypoxic conditions, HIFα is stable and accumulates, as the aforementioned hydroxylation and proteolyzation do not occur. The accumulated HIFα subsequently dimerizes with HIFβ constant region, and the heterodimer then moves into the nucleus to bind with hypoxia-response elements in the DNA. Finally, diverse genes related to cellular energy metabolism and angiogenesis are transcriptionally and translationally expressed [27]. Therefore, in ccRCC with *VHL* inactivation in which *VHL* does not function normally, the accumulation of HIFα occurs regardless of the oxygen status. In addition, the *VHL*–HIF pathway is further enhanced due to the effect of the hypoxic TME that occurs with tumor progression. It is well known that angiogenic growth factors, especially VEGF-A, are overexpressed via the *VHL*–HIF pathway in ccRCC [16,28]. VEGF exhibits pro-tumorigenic effects in addition to its effect in facilitating the vascular development of tumors: for example, VEGF stimulates VEGFR-2–JAK2–STAT3 signaling, which induces the self-renewal of cancer stem cells by upregulation of *MYC* and *SOX2* [29].

### 3.2. Anti-VEGFR and VEGF Inhibitors in ccRCC

Based on the preclinical studies discussed in the previous section, a therapeutic strategy aiming to block VEGF was attempted using bevacizumab, which is a monoclonal antibody for VEGF-A. In a first-line phase III trial of patients with mRCC, patients treated with bevacizumab in combination with IFNα showed longer progression-free survival (PFS) than patients treated with IFNα in combination with a placebo (hazard ratio [HR] 0.63, *p* = 0.001) [30]. However, despite the confounding effect of subsequent TKI therapy, the response did not seem to last long (HR of overall survival [OS] 0.91, *p* = 0.336) [31]. Studies targeting VEGFR were conducted in the direction of the broader inhibition of HIF-targeted molecules such as VEGFR-2 and PDGFR, as the insufficient potency of early TKI which is more specific to VEGFR itself [32]. A pivotal phase III study was a trial comparing sunitinib and IFNα: researchers observed a definite survival gain in patients treated with sunitinib relative to patients treated with IFNα (HR of PFS 0.42, *p* < 0.001), and subgroup analysis favored the use of sunitinib in all cases regardless of patient risk factors [33]. After the success of sunitinib, other TKIs, such as pazopanib, axitinib, and tivozantinib, demonstrated similar outcomes as first-line therapies for mRCC [34,35,36]. Cabozantinib, a TKI that targets multiple kinases, including MET, VEGFR, and AXL, increased survival and improved objective responses in patients with mRCC who were treated with one or more VEGFR TKIs [37,38]. In a phase II study, cabozantinib was found to increase PFS more than sunitinib in initial therapy for patients meeting the international mRCC database (IMDC) intermediate- or poor-risk population (HR 0.48, *p* = 0.0008) [39]. Since then, numerous preclinical and clinical data have been accumulated that provide strong evidence for the application of anti-angiogenic agents as major therapeutic options for mRCC.

### 3.3. Other Targets: The Protein Kinase B–Mechanistic Target of Rapamycin (AKT–mTOR) and Epidermal Growth Factor Receptor (EGFR) Pathways

*VHL* can inhibit mTORC1 signaling through degradation of regulatory associated protein of mTOR (RAPTOR) [40]. Thus, *VHL*-defective RCC enhances mTOR pathway activation. In addition, an increase in HIFα activates the AKT–mTOR pathway, which increases the survival of tumor cells in RCC [41]. Increased mTOR creates a vicious cycle that re-increases HIFα [41,42]; inhibition of the mTOR pathway was found to reduce HIF1α in a mouse model [43]. Consequently, studies have been conducted to apply mTOR inhibitors in the treatment of mRCC. A longer median OS was observed with temsirolimus treatment than with IFNα as first-line therapy for mRCC [44]. After anti-VEGFR therapy, treatment with everolimus expanded PFS compared to placebo [45]. 

SET and MYND domain-containing protein 3 (SMYD3) expression was elevated in *VHL*-defective RCC and cooperation between SMYD3 and SP1 increased EGFR expression and enhanced down-signals of EGFR [46]. The *VHL*-HIFα–EGFR pathway increases the proliferation and survival of RCC tumor cells through downregulation of RAF–MEK–ERK [47,48]. The combination of anti-EGFR and anti-VEGF was found to have anti-tumor activity through the regulation of the ARK and ERK signaling pathways [49]. Anti-EGFR therapy has been shown to inhibit tumor cell growth and angiogenesis in mouse models [49,50]. Thus, future studies on strategies for the effective use of anti-EGFR agents are likely to be beneficial.

### 3.4. Resistance to VEGFR Inhibitors: Alternative Anti-Angiogenic Pathways

The development of VEGFR inhibitors was a breakthrough in the era of systemic cytokine therapies for mRCC. However, unmet needs still remain in clinical practice. First, there are patient subpopulations, such as those in poor IMDC risk groups, for whom TKI therapies have unsatisfactory effects. Although various anti-VEGFR TKIs have been developed, when used as first-line therapies, clinical outcomes represented by median PFS remain approximately 8–10 months [51]. After resistance to anti-VEGFR TKIs develops, tumor vascularity often increases [52]. In addition, Casanovas et al. reported that a short-term VEGFR blockade triggered hypoxia and consequent upregulation of the FGF signaling pathway [52]. Therefore, the cause of this resistance and revascularization is likely an alternative angiogenic pathway independent of VEGF, such as the Ang–Tie pathway, IL-8, FGF, or PIGF [52,53]. However, the transcriptional expression of IL-8, FGF, and Ang are also regulated by hypoxia and HIF regulation [54,55]. Consequently, the HIF pathway is still important, even after the failure of anti-VEGFR therapy.

## 4. The TME and Anti-Cancer Immunity in ccRCC

Highly vascular ccRCC is characterized by *VHL* inactivation and hypoxic TME under conditions of excessive consumption of oxygen and nutrients [56]. These conditions allow the stabilization and accumulation of HIFα. HIF-mediated responses lead to alterations in immune cell activity and tumor metabolism [56]. The tumor microenvironment likely transitions to an immunosuppressive environment. However, a few reported cases of spontaneous regression of RCC and of durable remission with high-dose IL-2 therapy suggest that the TME is not always immunosuppressed in ccRCC [2,57]. Therefore, with the introduction of ICIs in mRCC treatment, a clearer understanding of the RCC TME is required.

### 4.1. Cellular Metabolism

PHD, an essential catalytic enzyme for the hydroxylation of HIFα, requires α-ketoglutarate as a co-substrate [58]. Ketoglutarate is metabolized to succinate, fumarate, and malate through the tricarboxylic acid cycle, which in turn is again metabolized to ketoglutarate [58]. This metabolism requires succinate dehydrogenase (SDH) and fumarate hydratase (FH), and alterations in genes encoding SDH and FH are common in ccRCC [59]. The dysfunction of SDH and FH causes the accumulation of succinate and fumarate and a shortage of ketoglutarate, eventually suppressing PHD activity [58]. As such, the cellular metabolism of ccRCC contributes to the stabilization of HIFα and further enhances the HIF pathway [56]. The enhanced HIF pathway then sustains and promotes glycolytic metabolism through the upregulation of glucose transporter 1 (GLUT-1) and induction of interferon gamma (IFNγ) [60]. As a result, the TME exhibits glucose depletion, lactate accumulation, and acidification [27], and anti-tumor immunity is likely weakened in ccRCC. For example, lactic acid, one of the metabolites produced by glycolysis, is associated with an increase in M2-tumor associated macrophages (TAMs), which have pro-tumoral effects in the TME [61]. In addition, acidification of the TME decreases T-cell activity through programmed death-ligand 1 (PD-L1) upregulation in TAMs [61]. 

Meanwhile, depletion of glucose causes glutamine addiction by using reductive carboxylation rather than oxidative metabolism [62,63]. Mitochondria protein sirtuin 4 (SIRT4) regulates glutamine metabolism through involvement in the conversion of glutamate to α-ketoglutarate in mitochondria [64]. A recent study reported that the SIRT4 induced metabolic stress through accumulation of ROS-induced apoptosis and clarified a regulatory mechanism between SIRT4 and *VHL*-HIF1α- Heme oxygenase-1 (HO-1) which is part of an endogenous defense system against oxidative stress [64]. Considering this changes of glycolytic and glutamine metabolism, a glutaminase inhibitor called telaglenastat (CB-839) has been developed and is under clinical trial in advanced ccRCC (NCT03428217).

### 4.2. Chronic Inflammation and TME

In *VHL*-defective RCC, the metabolic imbalance can cause chronic endoplasmic reticulum (ER) stress and unresolved ER stress induces chronic inflammation in RCC [65]. Kidney tissue of a *Vhl* conditional knockout mouse model exhibited epithelial disruption and interstitial inflammation [66]. This inflammatory response is mediated by overproduction of reactive oxygen species (ROS) through a lipocalin 2 (LCN2)-dependent manner in *VHL* inactivation [67]. In addition, endothelial cells (ECs) surrounding renal tubular epithelial cells show characteristics of inflammatory response, and cross talks between ECs and renal tubular cells are induced by oncostatin M (OSM), which activates ECs [68]. The activated ECs can recruit macrophages and induce polarization to M2-macrophages [68]. This will affect the composition of pro-tumorigenic microenvironment.

### 4.3. HIF, VEGF, and Immunosuppressive TME

The concept of tumor immunity has shifted from the past host-protection-centered immunosurveillance theory to immunoediting, which includes both host and tumor perspectives [69,70]. Chen et al. summarized the steps the immune system takes against cancer development in the cancer-immunity cycle, which is the process that leads to the recognition of cancer-associated antigen (CAA) by major histocompatibility complex (MHC) molecules on antigen-presenting cells (APCs), T-cell priming, education, and moving to TME, cancer-killing effects, the release of cytokines and CAA, and enhancement of anti-tumor immunity [71]. These processes can be skewed by diverse factors in both the host and the tumor. The enhanced HIF pathway of ccRCC is the one of those factors [56]. 

The ccRCC TME is rich in tumor-infiltrating lymphocytes (TILs) [72,73]. The percentage of cluster of differentiation (CD)3+ T cells was 69.7% and that of CD3+/CD8+ T cells was 42.6%, which was significantly higher than that in other subsets of T cells [72]. This can be inferred from a previous study that found that VEGF-A which had been induced by HIF-1α promoted tumor infiltration of CD8+ T cells through regulation of endothelium permeability and maintenance of the effector state of CD8+ T cells [74]. Two signals are required for the activation of T-cell: the first T-cell receptor (TCR) must recognize antigens presented by MHC molecules on APCs, and costimulatory signals must work [75]. The costimulatory molecule CD28 on T cells competes with inhibitory molecules to bind B7 ligands on APCs and tumor cells [75]. Thus, changes in MHC molecules and inhibitory molecules expressed on tumor cells affect the function of effector T cells. Doedens et al. reported that HIFα induced low expression of MHC-I molecules in tumor cells and that MHC-I expression was lower in *VHL*-defective RCC cells than in RCC cells with restored *VHL* [76]. In a conditional *VHL* inactivation murine study, T cell exhaustion was induced by the upregulation of inhibitory checkpoint molecules such as TIM-3, LAG-3, and CTLA-4 [77]. In addition, HIF1α inhibition was found to reduce PD-L1 expression in a mouse tumor model [78]. 

CD4+ T cells differentiate between helper T cells and regulatory T cells (Tregs) by inflammatory cytokines. Among these subsets, Tregs function in immune-suppressive activities through disturbance of interleukin-2 (IL-2) signaling, inhibition of IL-2 production, and expression of coinhibitory checkpoint molecules [75]. FOXP3+ Tregs selectively express VEGFR2 compared with FOXP3− Tregs [79], and HIF-1α induced the differentiation of T cells toward FOXP3+ [80]. These results suggest that FOXP3+ Tregs infiltrated the TME of ccRCC. Indeed, the expression of transforming growth factor-β (TGFβ)-mediated Tregs is associated with poor patient prognosis [81,82]. However, further elucidation of the impact of Treg on the ccRCC TME is needed, as one study reported a contrasting result in which HIF1α was found to drive IFNγ-induced Treg fragility [83]. 

In myeloid cells, HIF1α was found to induce the differentiation of TAMs to the M2-lineage and promote tumor angiogenesis by HIF1α-dependent matrix metallopeptidase 9 (MMP9) [84]. HIF1α upregulates PD-L1 expression on myeloid-derived suppressor cells (MDSCs) and suppresses overall T-cell activity [85,86]. 

Taken together, these studies suggest that the enhanced HIF pathway induces immunosuppressive TME in ccRCC. Although the ccRCC TME exhibits disturbances of immunosuppressive MDSCs, M2-macrophages, and Tregs, the induction of inhibitory checkpoint molecules such as PD-L1 on diverse subsets of immune cells is a promising attribute of the use of ICIs in the ccRCC treatment.

### 4.4. Immunomodulatory Effects of Anti-VEGF and Anti-VEGFR Therapies

Normalization of tumor vascularity is the main concept in anti-angiogenic therapy [87]. It alleviates the hypoxic TME state and facilitates more efficient drug delivery [88]. VEGFR blockade upregulates C-X-C motif chemokine ligand 10 (CXCL10), which is a cytokine related to the homing of immune cells into the TME [89,90] and promotes the intratumor infiltration of CD4+ or CD8+ T cells [91]. Huang et al. reported that lower doses of anti-VEGFR-2 antibody can reprogram the TME through redistribution of TAM and induction of infiltrating T cells than higher doses [92]. This result suggests that excessive destruction of tumor vessels by anti-VEGFR TKIs may hinder positive TME modulation by impairing the proper delivery of drugs and oxygen. Depending on the purpose of using TKIs, an appropriate dose can provide a favorable environment for the anticancer immune response. Additionally, anti-angiogenic agents downregulate the inhibitory checkpoint on CD8+ T cells and reverse T-cell exhaustion in the TME [93]. In relation to antigen presentation, pazopanib upregulated MHC molecules on dendritic cells and activated the maturation of dendritic cells by inhibiting the extracellular signal-regulated kinase (Erk)–β-catenin pathway [94]. Additionally, anti-angiogenic agents likely impact immune-suppressive cells in the TME. The blockade of VEGF-A reduced the proliferation of Treg cells in a murine study [95]. Sunitinib decreased Treg and MDSCs by inhibiting signal transducer and activator of transcription 3 (STAT3) [96]. Lenvatinib reduced TAM levels in a mouse tumor model [97]. Taken together, the blockade of the VEGF–VEGFR signaling can modulate the ccRCC TME toward an environment of more favorable anti-cancer immunity. This strongly supports the recent approach of using a combination of anti-angiogenic agents and ICI to treat ccRCC.

### 4.5. Immune Check Point Inhibitor-Combination Strategies

ICI as a treatment for ccRCC was introduced alongside nivolumab, an anti-PD1 monoclonal antibody. In 2015, a randomized phase III study of nivolumab and everolimus showed the probability of ICI application as a treatment for metastatic ccRCC (HR of OS 0.73, *p* = 0.002) [98]. A subsequent comparison study of first-line therapies in ccRCC patients with intermediate- or poor-IMDC risk found a survival gain in patients receiving nivolumab with ipilimumab, a CTLA-4 inhibitor, over those receiving only sunitinib (HR 0.63, *p* < 0.001) [99]. The survival benefit was sustained in further analysis, with a median follow-up of 32.4 months (HR 0.66, *p* < 0.0001) [100]. The ccRCC treatment paradigm has shifted from the earlier anti-angiogenic therapy to ICI. 

Another promising strategy is ICI combined with an anti-angiogenic TKI. Based on the immunomodulatory effects of anti-angiogenic agents, three randomized studies were published in 2019 [101,102,103]. Although two studies, which related to atezolizumab plus bevacizumab and avelumab plus axitinib, did not meet the co-primary endpoint due to insignificant differences in OS, treatment with ICI in combination with anti-angiogenic agents resulted in significantly longer patient PFS than did treatment with only sunitinib in all three studies [101,102,103]. The HRs of OS and PFS were 0.53 (*p* < 0.0001) and 0.69 (*p* < 0.001), respectively, in the study of pembrolizumab plus axitinib compared with sunitinib [103]. Consistent with the results of treatment with nivolumab plus ipilimumab, in an extended follow-up, a continued survival benefit was observed in treatment with pembrolizumab in combination with axitinib compared to sunitinib alone (HR of OS 0.68, *p* = 0.0003; HR of PFS 0.71, *p* < 0.0001) [104]. 

Efforts to identify optimal partners for combination regimens have continued, and two other combination studies have been published in 2021 [105,106]. A CheckMate 9ER study comparing treatment with nivolumab in combination with cabozantinib versus sunitinib alone reported a HR of PFS is 0.51 (*p* < 0.0001) an HR of OS of 0.60 (*p* = 0.001) [105]. Treatment with pembrolizumab in combination with lenvatinib also resulted in significantly longer PFS and OS than treatment with sunitinib alone in the CLEAR study [106]. Surprisingly, treatment with pembrolizumab in combination with lenvatinib exhibited a complete response rate of 16.1% and an objective response rate of 71%. Regardless of the IMDC risk group, ICI combination treatments showed consistently favorable efficacies compared to anti-VEGFR TKI treatment alone, and the differences in effect between ICI combinations and TKIs were more prominent in the poor IMDC risk population (HR of PFS in pembrolizumab plus lenvatinib = 0.18, 95% confidence interval 0.08-0.42) [106]. These results of phase III trials placed ICI combination treatment as the new standard treatment for metastatic ccRCC (Table 1). 

## 5. Outlook for the *VHL*–HIF Pathway in ccRCC Treatment

### 5.1. Return to HIFa

As discussed above, *VHL* inactivation, tumor hypoxia, and cell metabolism in ccRCC all enhance the HIF pathway. The importance of the HIF pathway demonstrated in preclinical studies fostered speculation that there would be differences in clinical outcomes in ccRCC patients treated with anti-VEGFR TKI according to HIF expression; however, in an analysis of patients treated with sunitinib, the expressions of HIF-1α and HIF-2α were not associated with improvements in clinical outcome [107]. Therefore, a therapeutic strategy targeting the HIF pathway itself has been proposed to overcome the therapeutic limitation of TKIs. HIF-dependent transcriptional targets consist of more than 500 diverse genes involved in angiogenesis, glycolysis, the cell cycle, and oxygen-sensing [108,109]. The complexity of these hypoxia-responsive genes makes it difficult to predict the effect of HIF inhibition downstream signals. HIF1α is more widely overexpressed in precancerous lesions and early-stage than in late-stage ccRCC; moreover, HIF1α is commonly expressed in most normal human tissues [110]. In addition, HIF1α has a role as a tumor suppressor in TME; for example, it plays a role in maintaining energy production through glycolysis in immune cells with anti-tumor activity, such as M1-macrophages [111]. Considering these points, it is necessary to be cautious about the inhibition of HIF1α. In contrast, HIF2α is observed in more limited and specific cell types, such as the embryonic development stage and adult vascular endothelial cells [110]. Overexpression of HIF2α is primarily expressed in high tumor-burden RCC, and HIF2α, but not HIF1α, induces cell death by reducing HIF-mediated transcription in *VHL*-defective RCC cells [112]. As such, HIF2α is predicted to exert oncogenic effects targeting genes such as VEGFA, FIK1, ANGPT1/Tie2, PDGFb, c-Myc, CXCR4 and MMP9l, which are related to angiogenesis, the cell cycle, and cell proliferation and metastasis [113,114,115,116,117,118]. Therefore, a more nuanced understanding of HIFα molecules has led to the development of HIF2α inhibitors.

### 5.2. HIF2a Inhibitors

The first HIF2α antagonist identified was PT2385, which showed a tolerable safety profile and efficacy in patients with heavily pretreated ccRCC [119]. However, PT2385 was restricted by dose-limited pharmacokinetics; consequently, Belzutifan (MK6482), a second-generation HIF-2α inhibitor with an improved pharmacokinetic profile, was developed and tested in a phase I clinical trial (NCT02974738) [120,121]. A phase III clinical trial comparing patients with metastatic ccRCC who had previously been treated with three or fewer systemic therapies treated with Belzutifan alone versus those treated with everolimus is ongoing (NCT04195750). In addition, a combination study using Belzutifan is also being conducted. A phase II trial of Belzutifan in combination with lenvatinib showed activity in mRCC with a 22% of objective response rate and a 64% disease control rate. Based on its efficacy, a phase III study comparing belzutifan in combination with lenvatinib versus cabozantinib alone is ongoing (NCT04586231). Clinical studies related to the triplet regimen, including pembrolizumab, are in progress (NCT04736706). To increase the treatment responses, various types of combination therapies will be attempted.

### 5.3. Other Targets: *VHL* Substrate Targets and Multi-Omics Approaches

Recent studies related to *VHL* substrates other than HIFα suggest novel therapeutic targets for ccRCC [122,123,124]. Zinc fingers and homeoboxes 2 (ZHX2), a *VHL* substrate, contributes to oncogenesis of ccRCC by inducing NF-κB activation [122]. Like HIFα, prolyl hydroxylation of ZHX2 allows *VHL* to recognize ZHX2, which is ubiquitinated and degraded [122]. Reintroduction of *VHL* increased the proteasomal degradation of ZHX2 in *VHL*-defective ccRCC cells [122]. Depletion of ZHX2 inhibited the growth of ccRCC cells with *VHL* loss [122]. Scm-like with four malignant brain tumor domains 1 (SFMBT1) is another target of *VHL* [123]. Prolyl hydroxylation of SFMBT1 also allows *VHL* to regulate its stability and depletion of SFMBT1 inhibited ccRCC cell growth [123]. In patients with ccRCC, the combined high expression of ZHX2 and SFMBT1 was significantly associated with prognosis [124]. In addition, recent advances in the clinical significance of radiomic profiling through tumor segmentation data from computed tomography, and understanding of genomic and metabolic subtypes, can lead to further improvements of treatments for ccRCC [125,126]. These multi-omics approaches will be useful in the development of prognostic and predictive biomarkers, as well as new therapies.

## 6. Closing Remarks

Although many years passed between the description of *VHL* disease and the discovery of the *VHL* gene, the understanding of the *VHL*–HIF pathway and its downstream networks has rapidly progressed. All advances in ccRCC have so far come with an improved understanding of these pathways. In addition, we anticipate that future improvements will take ccRCC therapies beyond the current standard of ICI in combination with anti-angiogenic agents, both leveraging and furthering our understanding of the *VHL*–HIF pathway.

## Figures and Tables

**Table 1:** Recent phase III findings on combination therapy in first line treatment of advanced RCC.

Study	Agents	N	Primary Endpoint	PFS (HR)	OS (HR)
CheckMate 214 ^99^ (NCT02231749)	1. Ipilimumab + Nivolumab	1096	PFS; OS; ORR	Met (0.85)	Met (0.71)
	2. Sunitinib				
JAVELIN Renal 101 ^102^ (NCT02684006)	1. Avelumab + Axitinib	886	PFS; OS	Met (0.69)	Unmet (0.78)
	2. Sunitinib				
IMmotion151 ^101^ (NCT02420821)	1. Atezolizumab + Bevacizumab	915	PFS; OS	Met (0.83)	Unmet (0.81)
	2. Sunitinib				
KEYNOTE-426 ^103^ (NCT02853331)	1. Pembrolizumab + Axitinib	861	PFS; OS	Met (0.69)	Met (0.53)
	2. Sunitinb				
CLEAR ^106^ (NCT02811861)	1. Pembrolizumab + Lenvatinib	1069	PFS	Met (0.39)	Met (0.66)
	2. Sunitinib				
CheckMate 9ER ^105^ (NCT03141177)	1. Nivolumab + Cabozantinib	651	PFS	Met (0.51)	Met (0.60)
	2. Sunitinib				

Abbreviations: HR, hazard ratio; N, number of enrolled patients; OS, overall survival; PFS, progression free survival; ORR, objective response rate.

## Data Availability

Not applicable.
